# Activity and post-prandial regulation of digestive enzyme activity along the Pacific hagfish (*Eptatretus stoutii*) alimentary canal

**DOI:** 10.1371/journal.pone.0215027

**Published:** 2019-04-05

**Authors:** Alyssa M. Weinrauch, Christina M. Schaefer, Greg G. Goss

**Affiliations:** 1 Department of Biological Sciences, University of Alberta, Edmonton, Alberta, Canada; 2 Bamfield Marine Sciences Centre, Bamfield, British Columbia, Canada; Universidade de Vigo, SPAIN

## Abstract

Hagfishes are living representatives of the earliest-diverging vertebrates and are thus useful for the study of early vertebrate physiology. It has been previously postulated that digestive enzymes account for the majority of digestion because hagfish are agastric with notable zymogen granules in specialized cells of the hindgut. While the presence of some digestive enzymes (amylase, lipase and leucinaminopeptidase) have been confirmed with histochemistry, quantification of enzymatic activity is limited. This study sought to biochemically quantify the tissue activity of six digestive enzymes (α-amylase, maltase, lipase, trypsin, aminopeptidase and alkaline phosphatase) along the length of the Pacific hagfish (*Eptatretus stoutii*) alimentary canal. In addition, the effect of feeding on the rate of enzyme activity was examined. Overall, maltase and trypsin activities were unchanging with respect to location or feeding status, while the activities of α-amylase and alkaline phosphatase decreased substantially following feeding, but were consistent along the length. Lipase and aminopeptidase activities were elevated in the anterior region of the alimentary canal in comparison to the more posterior regions, but were not altered with feeding. This study indicates hagfish have an assortment of digestive enzymes that likely are the result of a varied diet. The differential expression of these enzymes along the tract and in regards to feeding may be indications of early compartmentalization of digestive function.

## Introduction

Digestion is essential for the catabolism and hydrolysis of ingested macronutrients into smaller molecules suitable for transport. It is carried out using mechanical, chemical and enzymatic methods with digestive enzymes released from multiple locations along the alimentary canal. There are a multitude of digestive enzymes for each type of macronutrient, with specifications for substrate and optimal reaction conditions (*e*.*g*. acidic *vs*. alkaline), which correspond to their location in the digestive tract and can be derived from the stomach, exocrine pancreas, or the intestinal mucosa itself (reviewed in [1]). The capacity for an organism to digest certain foods predominantly depends upon the presence of appropriate enzymes [2]. The complement of digestive enzymes found in bony fishes are consistent with what is found in other vertebrates [3], however few reports exist focusing on hagfish.

The hagfishes are useful models for studies of evolutionary comparison as they are the oldest extant representatives of the first vertebrates [4]. They have a wide range of prey items consisting of both living and dead invertebrate and vertebrate species [5,6] and the consumption of decaying organisms provides the hagfish with a vital ecological role in the nutrient cycling and substrate turnover of the marine benthos [7]. The hagfish alimentary canal has been morphologically characterized with various microscopy techniques in both the Atlantic (*Myxine glutinosa*) and Pacific (*Eptatretus stoutii*) species [8,9]. With a structure unique to chordates, the mucous cells are contained solely within the foregut whereas the hindgut consists of a monolayered epithelium of both absorptive columnar cells and zymogen granule cells containing digestive enzymes [8]. Parallels have been drawn between the pancreatic tissue and the dispersed zymogen granule cells in the hagfish hindgut [10] and enzymatic activity in the hindgut has been examined, albeit solely on the Atlantic species, *M*. *glutinosa*. Adam (1963) [8] histochemically demonstrated the presence of amylase, lipase, and leucinaminopeptidase in all regions of the hindgut and postulated that feeding could induce changes in enzyme activity. In addition, Nilsson and Fänge (1970) have characterized the activities of trypsin, chymotrypsin, carboxypeptidase A, leucineaminopeptidase and a catheptic-type protease [11]. The question yet remains as to whether Pacific hagfish: (1) possess a full complement of digestive enzymes, (2) have differential enzyme distribution along the alimentary canal, and (3) modulate enzyme activity post-feeding. To address these queries we investigated the enzymatic activity for each class of macronutrient. α-amylase and maltase activity were assessed to determine carbohydrate digestion, as polysaccharides such as glycogen are obtained from the liver tissue of prey. α-amylase hydrolyses large polysaccharides into smaller molecules of maltose and glucose, whereas maltase yields glucose from maltose and is an excellent metric to assess complex carbohydrate digestion [12]. Lipid digestion was quantified by measure of lipase activity as it converts dietary lipids, such as fats and triglycerides, into monoglycerides and fatty acids [13]. Finally, protein digestion capacity was measured by trypsin, aminopeptidase and alkaline phosphatase activity. Trypsin is a digestive protease produced in the pancreas, while aminopeptidase is derived from the small intestine and cleaves individual amino acids from proteins [14,15]. Alkaline phosphatase operates at an alkaline pH and has numerous physiological roles including mediation of inflammation, nutrient absorption and maintenance of intestinal pH [16]. We hypothesized that the varied diet of hagfish, from the glycogen- and lipid-rich liver tissue [17] to proteinaceous muscle tissue [5], would necessitate a full complement of digestive enzymes that would have elevated activity in the hindgut compared to the anterior alimentary tract (buccal cavity (B) and pharyngocutaneous duct (PCD)) owing to the presence of zymogen granule cells. Additionally, we expected reduced rates of tissue enzyme activity following feeding, as the digestive contents of the zymogen granule cells would have been released at the onset of feeding. Overall, we were able to quantify the activity of each investigated enzyme. Each enzyme had a distinct expression profile along the length of the alimentary canal, and a variable response to feeding, which may be indicative of early compartmentalization of gut function.

## Materials and methods

Twenty-four Pacific hagfish (*Eptatretus stoutii*; 65.3 ± 3.5 g; mean ± standard error of the mean (s.e.m)) were collected using traps baited with hake (*Merluccius merluccius*) in Trevor Channel, Bamfield, B.C., Canada (N48°50.883-W125°08.380) under a license approved by the Department of Fisheries and Oceans Canada (permit No. XR-136-2017). The animals were immediately transferred to ~5000 L holding tanks with continuously flowing seawater at Bamfield Marine Sciences Station, prior to shipping to the University of Alberta where they were housed in a recirculating artificial salt water system (Instant Ocean SeaSalt; Spectrum Brands, Blacksburg, VA, USA). This 2400 L system is constantly circulated through 6 tanks in a flow-through manner and is maintained at 12 ± 2 °C and 24 ± 2 ppt salinity. Owing to their light sensitivity, hagfish were housed in blackened containers at all times with PVC piping used as habitat enrichment as previously described [17]. Much like some reptiles, hagfish are intermittent feeders, known to regress intestinal function and cellular morphology between feeding periods [17]. It is not uncommon for hagfish to ignore food between feedings for multiple weeks at a time (personal observation), so we opted for a one-month fasting period to mirror natural fasting periods of this animal. Fed animals were given squid, permitted to feed until satiated and to digest for a 2 h period. Previous experiments have demonstrated that heightened physiological perturbations, such as metabolic oxygen consumption, occur 8 h after a feed [17]. We opted to use a 2 h post-fed time frame to examine the tissue enzyme activity near the onset of digestion, rather than at the peak point of many physiological processes to increase the likelihood that tissue enzyme activity would persist post-feeding. All sampling procedures and experimental manipulations were conducted with the approval of the University of Alberta Animal Care Committee (No. AUP0001126 (2017)).

### Tissue preparation

Hagfish were euthanized by an overdose of tricaine methanesulfonate (TMS; 5 g L^-1^; Syndel Laboratories Ldt., Nanaimo, B.C., Canada) in artificial seawater. The animals were dissected along the mid-ventral line and the digestive tract was excised and gently flushed with 0.5 M NaCl to clear contents. The animals were eviscerated to ensure death. The digestive tract was then divided into 5 equal portions as follows: buccal cavity (B), pharyngocutaneous duct (PCD), anterior hindgut (Ant HG), mid hindgut (Mid HG), and posterior hindgut (Post HG; see S1 Fig). The tissues were immediately placed in homogenization buffer (50 mM imidazole, 2 mM EDTA; pH 7), homogenized on ice (Polytron PT 1200 E; Kinematica AG, Lucerne, Switzerland) and stored in aliquots at -80 °C until biochemical analysis. Of note, luminal contents were also collected and analysed. However, the amount of activity detected was orders of magnitude below that detected in the tissue due to dilution by the digestive fluid and the food itself. For this reason, no further analysis of luminal content activity was conducted or reported herein.

### Enzymatic assays

All assays of digestive enzymatic activity were carried out as previously described. The tissue activity of each enzyme was measured in each portion of the digestive tract and compared to a substrate only blank to account for endogenous product in that solution. Samples were read on a microplate spectrophotometer (Spectromax 190, Molecular Devices, Sunnyvale, CA, USA) using clear, flat-bottom 96-well microplates. Unless otherwise noted, all chemical compounds, reagents and enzymes were supplied by Millipore-Sigma (St. Louis, MO, USA).

#### α-amylase activity

α-amylase activity was measured according to the Somogyi-Nelson method [18,19], with modifications [20]. Briefly, solution 1 (53.2 mM sodium potassium tartrate, 283 mM sodium carbonate, 238 mM sodium bicarbonate, 1.27 M sodium sulfate) and solution 2 (80 mM copper sulphate pentahydrate, 1.27 M sodium sulfate) were prepared and stored separately in brown glassware. Solution 1 and 2 were mixed (4:1) to create a working reagent immediately prior to analysis. Homogenate (60 μL), working reagent (60 μL) and substrate (60 μL of 1% starch boiled in 0.8 M sodium citrate buffer; pH 7) were combined for 20 min. Following incubation, Nelson reagent (60 μL of 0.28 M ammonium molybdate, 0.38 M sodium arsenate dibasic, 21 mL concentrated sulphuric acid—stored at 37 °C for 24 h in brown glassware) was added and the reaction proceeded for 15 min before termination with 1 μL of 1 M HCl. Samples were centrifuged (2 min @ 14, 000 *x* g), plated, read at A600 nm and compared to a glucose standard curve. α-amylase activity was expressed as nmol glucose liberated min^-1^ mg protein^-1^.

#### Maltase activity

Maltase activity was determined as previously [21]. The homogenate (50 μL) was combined with substrate (50 μL of 62.5 mM maltose) and incubated for 1 minute. The reaction was terminated with 1 μL of 1 M HCl and the samples were centrifuged (2 min @ 14, 000 *x* g). Following an incubation period of 5 min, glucose content was determined by combining supernatant (10 μL) with glucose cocktail (200 μL of 0.22 g MgCl_2_, 0.05 g NAD, 0.05 g ATP, 2.4 U/mL glucose-6-phosphate dehydrogenase in 50 mL triethanolamine hydrochloride; pH 7.5). The difference at A340 nm before and 15 min after the addition of hexokinase (5 U/ sample) was recorded and compared to a glucose standard curve. Maltase activity was expressed as nmol glucose liberated min^-1^ mg protein^-1^.

#### Lipase activity

Lipase activity was measured using modified methods [22]. The homogenate (6 μL) was incubated for 15 minutes in 86 μL of solvent (5.2 mM deoxycholic acid in 250 mM tris-HCl; pH 7.5) and 2.5 μL of 10 mM ethanol. The substrate (5.5 μL of 20 mM *p*-nitrophenyl myristate dissolved in ethanol) was added to the homogenate mixture and incubated for 15 min before centrifugation (2 min @ 6100 *x* g). Samples were read at A405 nm and compared to a *p*-nitrophenol standard curve. Lipase activity is expressed as μmol *p*-nitrophenol liberated min^-1^ mg protein^-1^.

#### Trypsin activity

Trypsin activity was measured as previously [23] wherein the homogenate (100 μL) was activated by enterokinase (4 U mL^-1^ in 40 mM succinate buffer; pH 5.6) for 15 min. The substrate (350 μL of 2 mM Na-benzoyl-l-arginine-*p*-nitroanilide hydrochloride (BAPNA) dissolved in DMSO (0.1%) in 100 mM tris-HCl; pH 8) was added to the activated homogenate (50 μL) and incubated for 1 h at 15 °C. The reaction was terminated with 100 μL of 30% acetic acid and the samples centrifuged (2 min @ 14,000 *x* g) and read at A550 nm against a *p*-nitroaniline standard curve. Activity was expressed as nmol *p*-nitroaniline liberated min^-1^ mg protein^-1^.

#### Aminopeptidase activity

Aminopeptidase activity was measured as previously [24]. Homogenate (30 μL) was combined with substrate (80 μL of 2.04 mM l-alanine-*p*-nitroanilide HCl in 200 mM sodium phosphate buffer; pH 7) and incubated for 15 min. Following centrifugation (30 s @ 14,000 *x* g) the samples were plated, read at A410 nm and compared to a *p*-nitroaniline standard curve. Final activity was expressed as nmol *p*-nitroaniline produced min^-1^ mg protein^-1^.

#### Alkaline phosphatase activity

Measurement of alkaline phosphatase activity was conducted as previously [25]. Homogenate (25 μL) was combined with substrate (55 μL of 20 mM *p*-nitrophenyl phosphate dissolved in 100 mM ammonium bicarbonate buffer containing 1 mM MgCl_2_; pH 7.8) and incubated for 15 min. Following centrifugation (30 s @ 14,000 *x* g), the samples were plated, read at A405 nm and compared to a *p*-nitrophenol standard curve. Aminopeptidase activity was expressed as μmol *p*-nitrophenol liberated min^-1^ mg protein^-1^.

#### Protein assays

All protein assays were conducted using commercial kits (bicinchoninic assay (BCA) or Bradford’s reagent for amylase assays) according to the manufacturer’s instructions (Thermo Fisher Scientific; Waltham, MA, USA).

### Statistical analysis

Datasets were first analysed using a Kruskal-Wallis 1-way analysis of variance (ANOVA) on ranks to discern if differences occurred between the anterior digestive tract (B and PCD) and the posterior digestive tract (anterior, mid and posterior hindgut). If a significant difference was found between these sections, the dataset was analysed independently using a 2-way ANOVA in each section (effect of feeding and effect of location). The sole exception was for lipase activity. Lipase activities in the anterior sections were not of equal variation and so an ANOVA could not be utilized. In this case, we analyzed the B and PCD separately using the Mann-Whitney Rank Sum Test. If no difference was detected between the anterior and posterior sections, a 2-way ANOVA was run on the entire tract length. Significance was accepted at α = 0.05 for all tests. In the instances where significant differences were detected, all pairwise multiple comparisons were made using a Bonferroni t-test post hoc analysis. All statistical analyses were conducted in SigmaPlot ver. 14 (Systat software Inc, integration, San Jose, CA, USA). Datasets were graphed using Prism 6 (GraphPad Software INC., La Jolla, CA, USA). All relevant data are within the paper and its Supporting Information files.

## Results

For each of α-amylase, lipase, trypsin and alkaline phosphatase, the anterior and posterior sections differed substantially (see S1 Table; H_1_ = 49.4, P <0.001; H_1_ = 9.36, P < 0.002; H_1_ = 58.4, P < 0.001; H_1_ = 68.4, P < 0.001). Therefore, the effect of feeding and location was examined independently in each section for these enzymes *via* a 2-way ANOVA or Mann-Whitney Rank Sum Test (see methods). Notably, the anterior region (B and PCD) was not analyzed for α-amylase, trypsin or alkaline phosphatase owing to the activities being below detectable limits. All other enzymes were analyzed using a 2-way ANOVA taking all sections into consideration.

**Fig 1:**
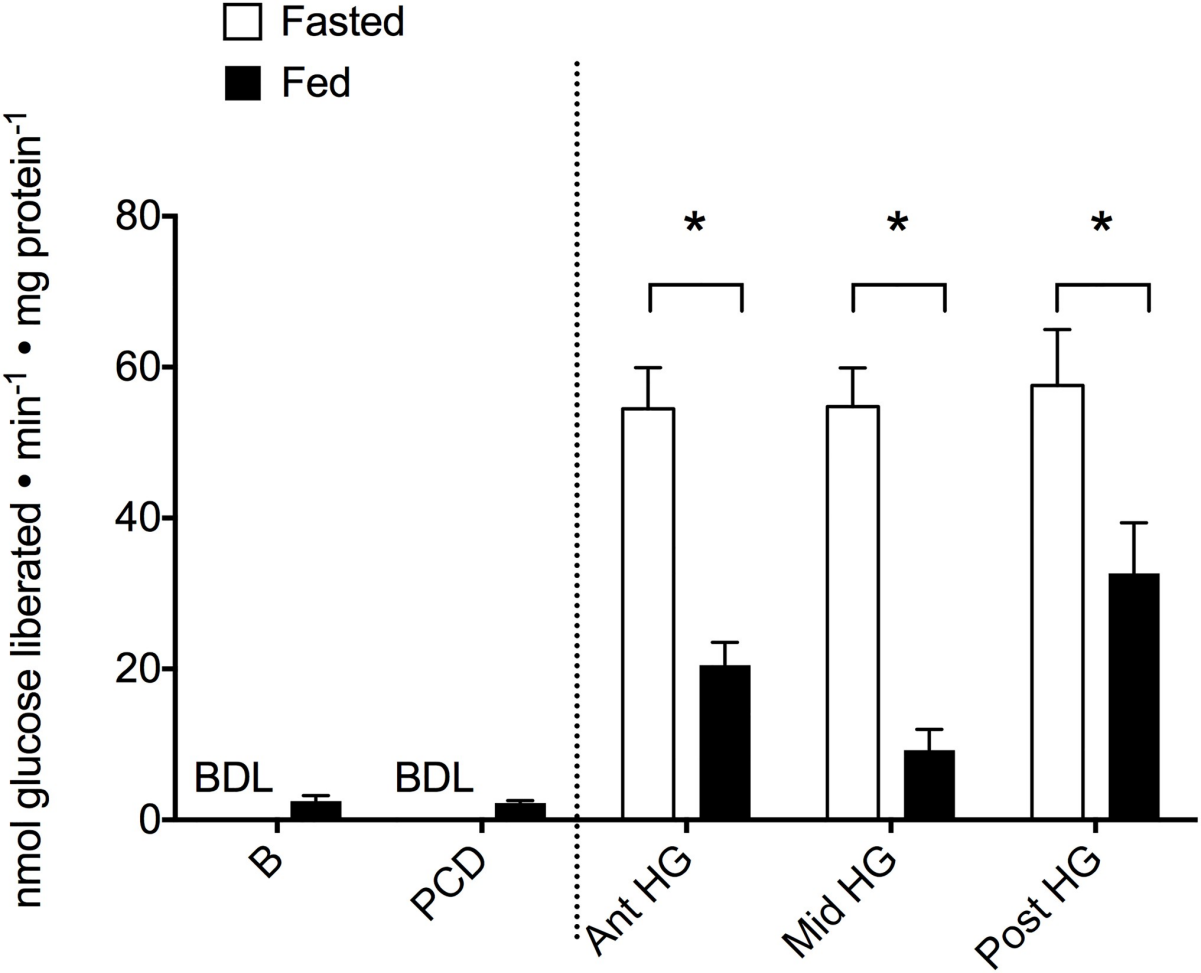
Changes in α- amylase activity (nmol glucose liberated min^-1^ mg protein^-1^) along the length of the Pacific hagfish alimentary canal and with respect to feeding status. Activity was measured in both fasted (white bars) and fed (black bars) hagfish in five locations along the alimentary canal (B—buccal cavity, PCD—pharyngocutaneous duct, Ant HG—anterior hindgut, Mid HG—mid hindgut, Post HG—posterior hindgut). Bars represent means + s.e.m. of 5–8 hagfish. A Kruskal-Wallis analysis determined that there were significant differences between the anterior and posterior segments of the tract, thus a 2-way ANOVA to determine effect of feeding and location was conducted in the hindgut only (right of the dotted line). Asterisks (*) denote significant differences with significance accepted at α = 0.05.

**Fig 2:**
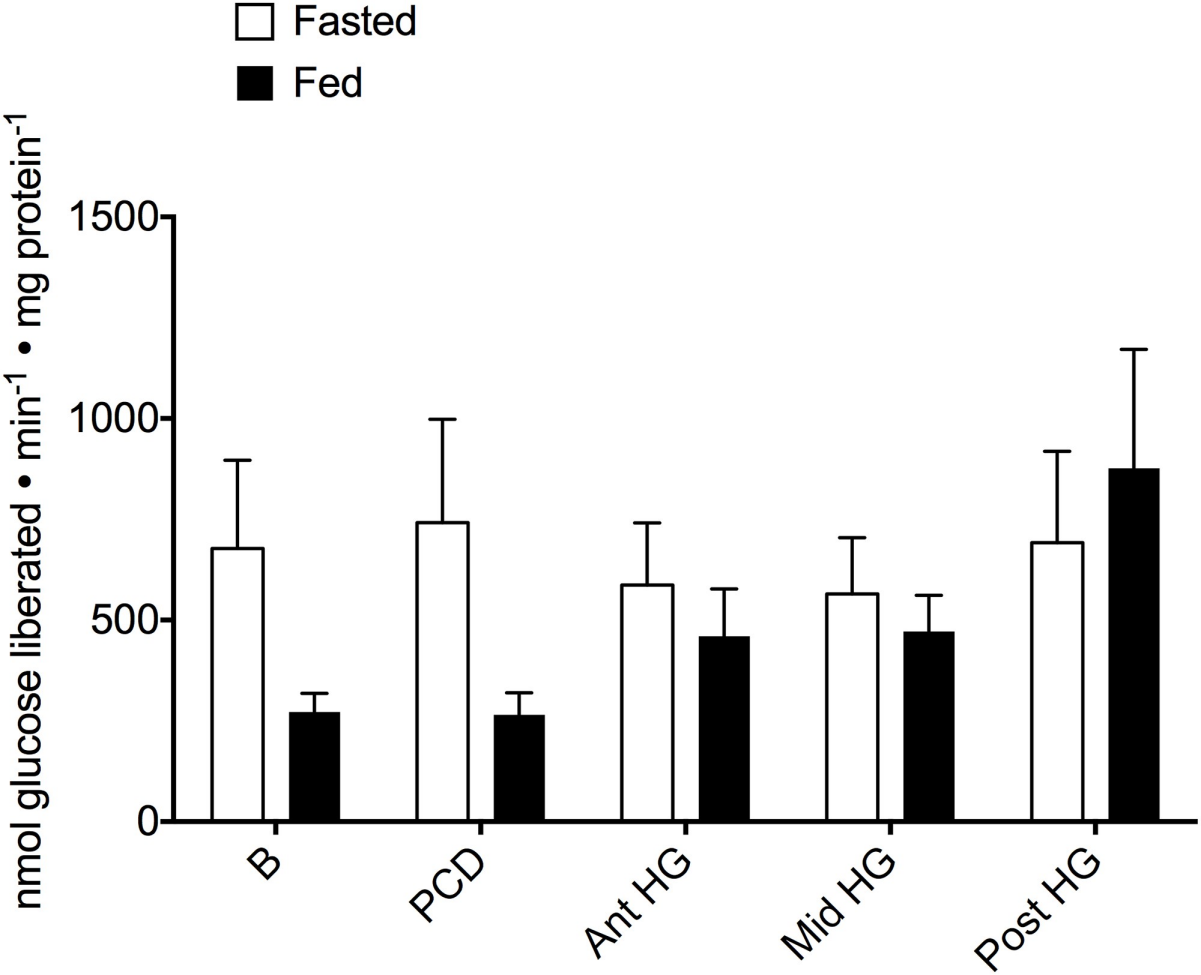
Maltase activity (nmol glucose liberated min^-1^ mg protein^-1^) does not change with feeding or location in the Pacific hagfish alimentary canal. Activity was measured in both fasted (white bars) and fed (black bars) hagfish in five locations down the alimentary canal (B—buccal cavity, PCD—pharyngocutaneous duct, Ant HG—anterior hindgut, Mid HG—mid hindgut, Post HG—posterior hindgut). Bars represent means + s.e.m. of 4–7 preparations. A 2-way ANOVA of the entire tract determined there were no significant effects of feeding or location (α = 0.05).

α-amylase activity was either minimal (fed fish) or below detectable limits (fasted fish) in the anterior portion of the digestive tract (B and PCD) however, the activity was significantly higher in all tested regions of the hindgut in both feeding states. Feeding significantly lowered the hindgut activity of α-amylase (Fig 1 and S2 Table; 37.4%, F_1,40_ = 56.038, P < 0.001). No significant differences were observed in maltase activity, either in regard to location or feeding status (Fig 2 and S2 Table; feeding: 71.7%, F_1,62_ = 2.826 P = 0.099; location: F_4,62_ = 0.926 P = 0. 456).

**Fig 3:**
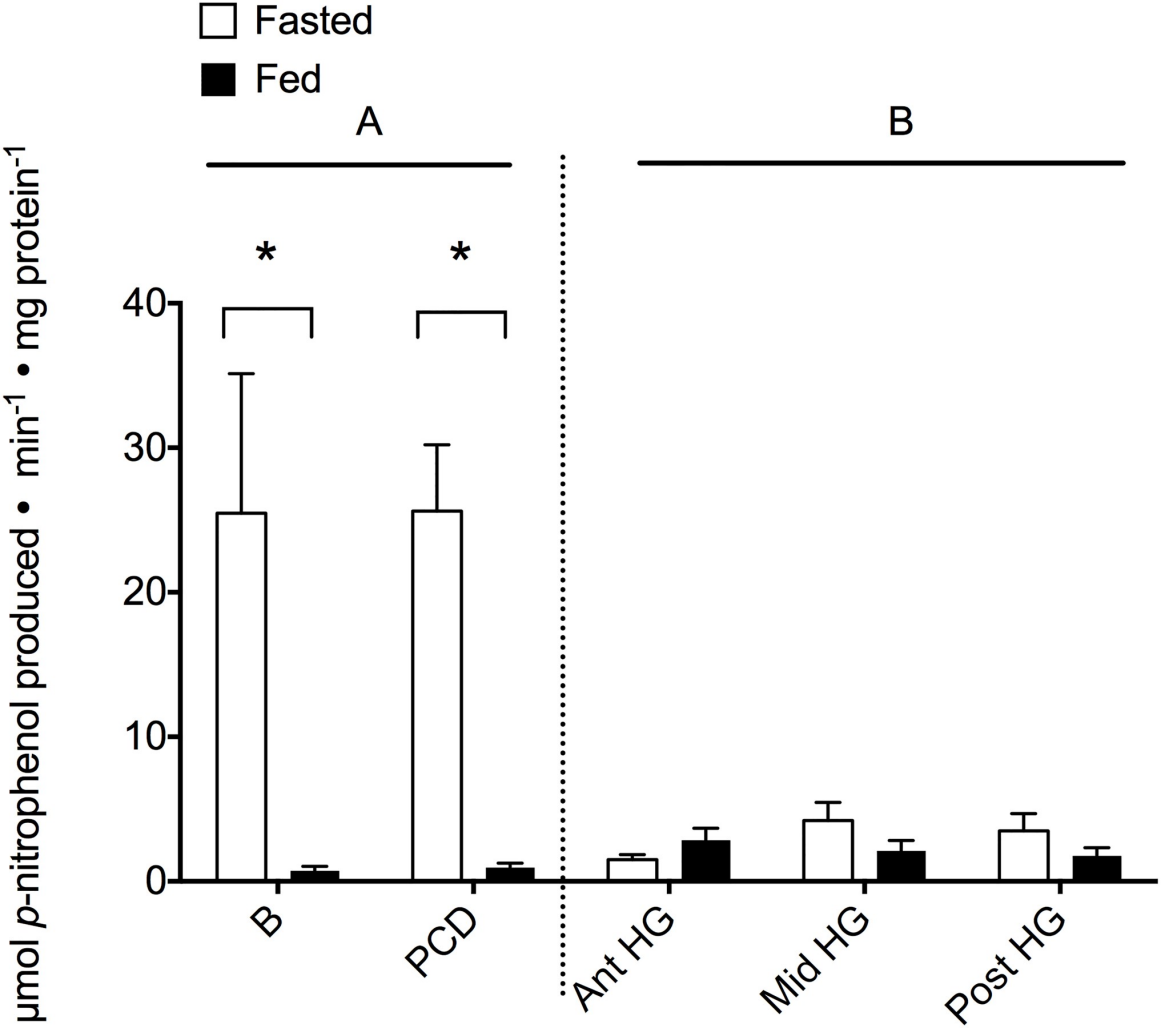
Lipase activity (μmol *p*-nitrophenol min^-1^ mg protein^-1^) is dependent upon location within the alimentary canal and significantly decreases post-feeding in the anterior segment. Activity was measured in both fasted (white bars) and fed (black bars) hagfish in five locations down the alimentary canal (B—buccal cavity, PCD—pharyngocutaneous duct, Ant HG—anterior hindgut, Mid HG—mid hindgut, Post HG—posterior hindgut). Bars represent means + s.e.m. of 5–11 hagfish. Letters denote significant differences between locations as determined by a Kruskal-Wallis (α = 0.05) comparing anterior *vs*. posterior segments (separated by the dotted line), whereas asterisks (*) denote difference in feeding state as determined using a Mann Whitney Rank Sum Test (α = 0.05) within a segment.

Lipase activity was detected at all points along the alimentary canal and was significantly elevated in the anterior segments (B and PCD) in comparison to the posterior segments (ant-, mid- and post-HG; Fig 3 and S2 Table; H_1_ = 4.11, P = 0.043). There was no effect of either feeding or location in the posterior location (feeding: 73.1%, F_1,50_ = 1.172, P = 0.285; location: F_2,50_ = 0.642, P = 0.531). Differences between feeding state were found for both the B and PCD (P < 0.001).

**Fig 4:**
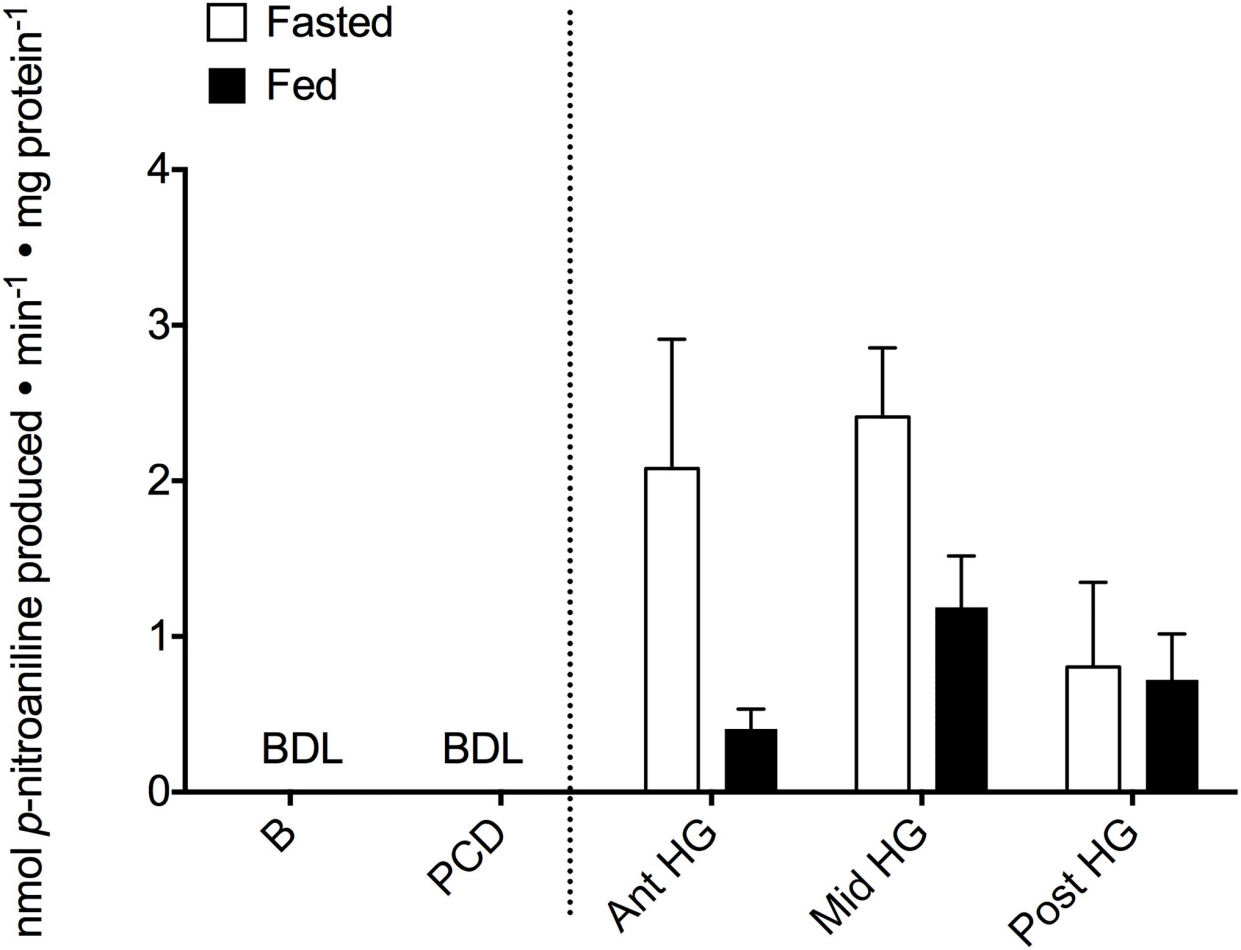
The trypsin activity (nmol *p*-nitroaniline produced min^-1^ mg protein^-1^) along the entirety of the Pacific hagfish hindgut does not change with feeding status. Activity was measured in both fasted (white bars) and fed (black bars) hagfish in five locations down the alimentary canal (B—buccal cavity, PCD—pharyngocutaneous duct, Ant HG—anterior hindgut, Mid HG—mid hindgut, Post HG—posterior hindgut). Bars represent means + s.e.m. of 3–8 hagfish. BDL = below the detectable limits of the assay. A Kruskal-Wallis analysis revealed significant differences between the anterior and posterior segments of the tract, thus a 2-way ANOVA to determine effect of feeding and location was conducted in the hindgut only (right of the dotted line). Significance was accepted at α = 0.05.

**Fig 5:**
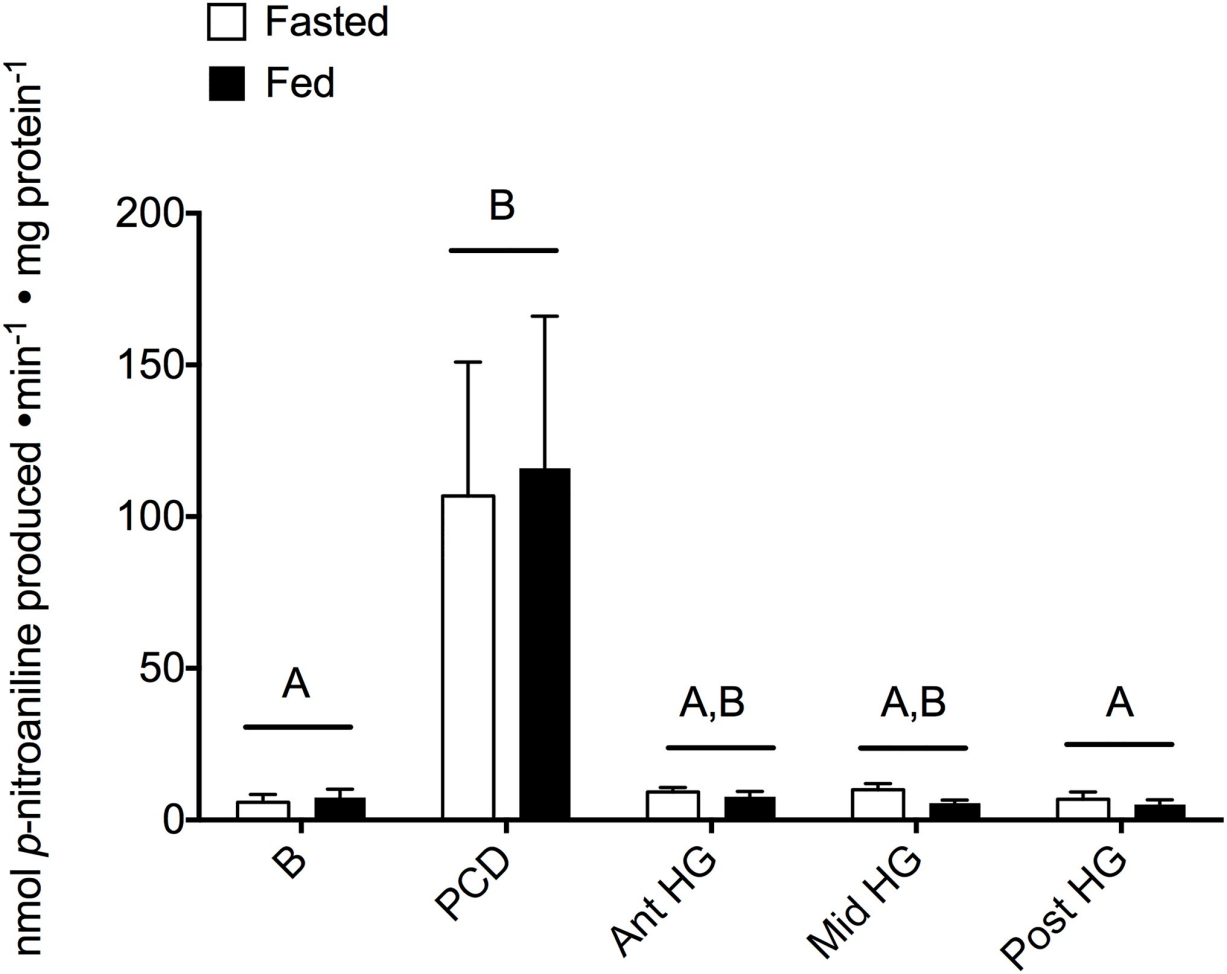
The activity of aminopeptidase (nmol *p*-nitroaniline produced min^-1^ mg protein^-1^) varies with location along the Pacific hagfish alimentary canal. Activity was measured in both fasted (white bars) and fed (black bars) hagfish in five locations down the alimentary canal (B—buccal cavity, PCD—pharyngocutaneous duct, Ant HG—anterior hindgut, Mid HG—mid hindgut, Post HG—posterior hindgut). Bars represent means + s.e.m. of 5–12 hagfish. A Kruskal-Wallis determined that there were significant differences in activity between different locations, with differences indicated by different letters. Significance was accepted at α = 0.05.

**Fig 6:**
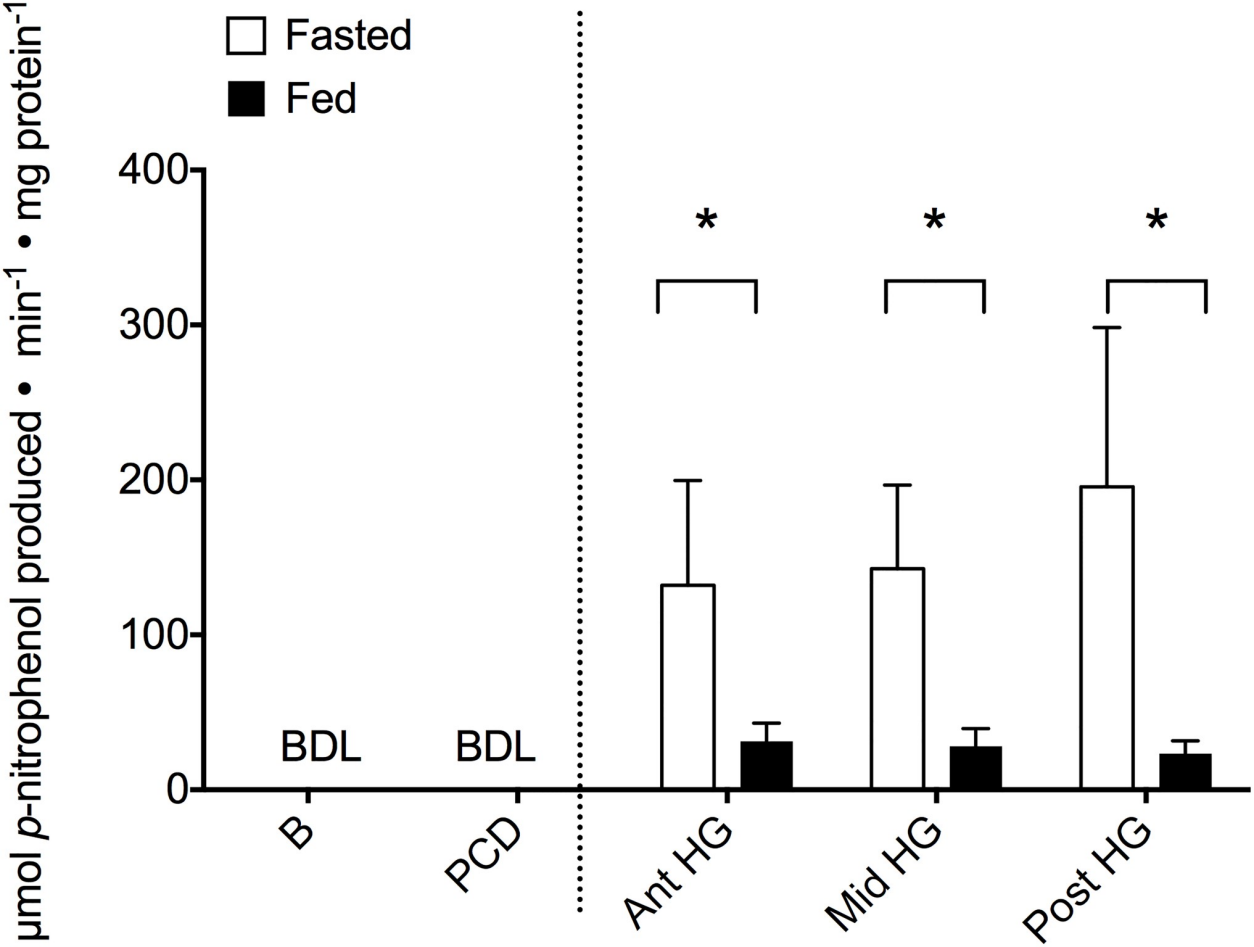
Feeding alters alkaline phosphatase activity (μmol *p*-nitrophenol produced min^-1^ mg protein^-1^) within the entire hagfish hindgut. Activity was measured in both fasted (white bars) and fed (black bars) hagfish in five locations down the alimentary canal (B—buccal cavity, PCD—pharyngocutaneous duct, Ant HG—anterior hindgut, Mid HG—mid hindgut, Post HG—posterior hindgut). Bars represent means + s.e.m. of 6–12 preparations. No activity was detected in the anterior portion of the alimentary canal (BDL = below detectable limits). Therefore, a 2-way ANOVA was conducted for the hindgut regions alone (right of the dotted line) with significance accepted at α = 0.05. Asterisks (*) denote significant differences between feeding states.

Trypsin activity was not detected in the anterior alimentary canal in either feeding state (Fig 4). The hindgut had detectable activity although there were no significant effects of feeding status or location within the hindgut (S2 Table; feeding: 57.1%, F_1,34_ = 2.19, P = 0.149; location: F_2,34_ = 0.675, P = 0.517). Aminopeptidase activity was most prominently expressed in the PCD however, no effects of feeding were observed at any location (Fig 5 and S2 Table; H_4_ = 13.2, P = 0.010). Finally, alkaline phosphatase activity was not discernable in the anterior regions of the tract (B and PCD) however, significant decreases in hindgut enzyme activity occurred following a feeding event (Fig 6 and S2 Table; 17.5%, F_1,45_ = 6.643, P = 0.014).

## Discussion

Overall, *E*. *stoutii* have digestive enzymes that catabolize each of the major macronutrient classes (carbohydrates, fats, proteins). Much like stomachless teleosts, the lack of a stomach does not appear to impact digestive flexibility or capacity in hagfish [26,27]. Table 1 summarizes the statistical relationships of tissue digestive enzyme activity between anterior and posterior segments of the hagfish alimentary canal. In the event of a significant difference between regions, a secondary effect within an applicable segment is presented. Each enzyme has a unique distribution along the alimentary canal (Table 1) and, for the most part, it seems that the majority of digestive activity takes place within the hindgut as previously suggested [8]; however, some digestive activity (maltase, lipase and aminopeptidase) was noted in the anterior regions (B and PCD). The typical vertebrate observation of decreasing enzyme activity in the posterior-most segments [28,29] was not evident along the hindgut of hagfish for any studied enzyme. Table 2 highlights the statistical outcome of feeding on digestive enzyme tissue activity in the Pacific hagfish, where differential effects of feeding were observed for certain enzymes.

**Table 1:** Summary table depicting statistical relationships for the localization of α- amylase, maltase, lipase, trypsin, aminopeptidase, and alkaline phosphatase tissue activities in the Pacific hagfish alimentary canal.

Enzyme	Anterior *vs*. Posterior		
		Within Anterior	Within Posterior
α-amylase	++		−
Alkaline phosphatase	++		−
Trypsin	++		−
Lipase	+	−	−
Aminopeptidase	− *		
Maltase	−		

+ significance *p* < 0.05

++ significance *p* < 0.01

− no significance

* difference detected along length as a whole

**Table 2:** Summary table of the effect of feeding on α- amylase, maltase, lipase, trypsin, aminopeptidase, and alkaline phosphatase tissue activities in the Pacific hagfish alimentary canal.

Enzyme	Effect of feeding
α-amylase	++
Alkaline phosphatase	+
Trypsin	−
Lipase	++
Aminopeptidase	−
Maltase	−

+ significance *p* < 0.05

++ significance *p* < 0.01

− no significance

The differential distribution and retention of some enzymatic function post-feeding supports the previous hypothesis that hagfish must maximize digestive function in their relatively straight and simple alimentary canal [30]. Owing to the difficulties in making quantitative comparisons with other species because of differences in methodology, reported units of activity, ontogenetic stages, and the amount and composition of the diet [31], the digestive enzymes in this study were not directly compared to other calculated activities.

### Digestive enzyme activity

It has long been known that carbohydrates are the preferred metabolic fuel of the hagfish [32,33]. Hagfish preferentially feed upon the glycogen-rich liver when presented with a whole carcass in captivity [17]. The digestive capabilities of polysaccharides have been investigated previously with α-amylase histochemically detected [8] and maltase activity expressed [34] uniformly across the hindgut. This study has confirmed that α-amylase is essentially restricted to the hindgut regions of the hagfish digestive tract and is uniformly expressed along its length. This uniform expression could be the result of the unchanging morphology along the hagfish hindgut [8,9], as well as the previously postulated hypothesis that hagfish maximize nutrient uptake along the hindgut length as there is no organ for food storage (i.e. stomach), and meals may be infrequent [30]. Indeed, glucose acquisition rate is consistent along the hindgut length of Pacific hagfish [34], demonstrating an equal role for digestive/absorptive processes along its length. Given that our results demonstrate a significant decrease in activity post-feeding (Fig 1), it is likely that amylase is contained within the digestive zymogen granule cells. The decreased tissue expression post-feeding therefore corresponds to the release of amylase granules at the commencement of digestion, much like that of vertebrate salivary glands. Indeed, the zymogen granule cells have been likened to both salivary and pancreatic glands of vertebrates [8,10]. Given that α-amylase is a primary protein that is stored in a bound zymogen granule prior to export from vertebrate salivary glands and pancreatic secretions [35,36], it is not surprising to find α-amylase activity in the hindgut containing a similar structure. Furthermore, the distribution of α-amylase activity coincides with the location of the zymogen granule cells, which supports this hypothesis that amylase is housed within the zymogen cells. Interestingly, α-amylase activity has also previously been detected around the slime glands and also the skin [8], which is known to acquire nutrients in hagfish [37–40]. It has previously been suggested that the slime possesses digestive activity thereby providing a means of external digestion while the prey is encased in a slime cocoon [34]. This hypothesis, however, requires further examination. Finally, previous characterization of hagfish α-amylase suggests a relatively elevated optimum pH of 8–9 [8], despite typical optima in the range of pH 6–8 [37]. This is puzzling as there are indications that hagfish acidify the hindgut lumen upon feeding [11] and the zymogen granule cells react positively to acidophilic stains [8,9]. However, α-amylase activity is known to differ depending upon food item [38]. Therefore, since the previously recorded optimal pH of hagfish α-amylase was determined *in vitro*, it is possible that an *in vivo* study may yield different results, particularly if in fact the lumen does become relatively acidic (pH 5.5–6.3) even in a fasting condition [11,30].

The recorded maltase activity was comparable to previously calculated rates in fasted *E*. *stoutii* (~ 200–1000 nmol min^-1^ mg protein^-1^; [34]). Similarly, the rate of activity was unchanging along the length of the alimentary canal, regardless of the nutritional state of the hagfish (Fig 2); features also found in the New Zealand hagfish (*E*. *cirrhatus*; [39]). This is comparable to the unchanging rates of amylase activity that reflect the natural diets of some teleosts, particularly herbivorous fish that feed relatively frequently [31,40–43]. However, most carnivorous teleost fish have increased maltase activity following a feeding event [44]. In the case of hagfish, the immutable rates of maltase activity would ensure a continual ability to digest this favoured macronutrient [33] and maximize uptake along the entire tract [34] when polysaccharides become available. Similar reports exist for some fish species and are suggested to be effective means to maximize nutrient assimilation along a relatively short tract with a rapid transit rate [27]. The differential expression and regulation of these polysaccharide-digesting enzymes is in accordance with their distinct substrate requirements. The zymogen release of α-amylase will cleave the larger polysaccharides into short and linear branched chains, with the final and pivotal transformation into the transported glucose conducted by maltase. Unlike amylase activity there is no change in post-fed tissue activity and maltase is found in the foregut as well as the hindgut, which suggests that maltase is stored differently than amylase. Vertebrate maltase is known to be a membrane-bound enzyme [45], and considering the continual expression in this study, membrane-bound maltase is a possibility for hagfish as well. Future studies should utilize imaging techniques (such as immunocytochemistry) to determine the specific localization of each of these enzymes. For this study, we mined both the published unannotated *E*. *burgerii* genome (GenBank assembly accession: GCA_900186335.2) and an Illumina transcriptome of *E*. *stoutii* to discern whether we had sequence conservation with commercially available antibodies for each of these digestive enzymes. Unfortunately, the degree of conservation was not satisfactory. The development of hagfish-specific antibodies would be a useful endeavour to confirm our hypotheses of digestive enzyme location.

Lipids are an invaluable energetic source for hagfish, particularly during extended periods of starvation, which can persist at least 11 months in captivity [32,46]. Recent evidence has suggested that hagfish have a regulated and specific mechanism by which lipids can be acquired in the hindgut [47] and lipases are essential for the digestion and assimilation of dietary lipids. There were significantly elevated lipase activities in the anterior tract (B and PCD) in comparison to all regions of the hindgut (Fig 3). Prominent lipase expression has been observed in the anterior digestive tracts of a number of teleost species and maximizes liberation of absorbable lipids along the rest of the tract [37,41,48,49]. We saw a significant reduction in anterior lipase activity following a feeding event, suggestive of enzyme release in the foregut regions. As fats are an invaluable energy source that require more time for digestion, this significant anterior release of lipase is likely responsible for the initial digestion prior to even reaching the hindgut where absorption occurs [47]. The decreased lipase tissue activity in the hindgut may be the result of various endogenous sources of lipase. Indeed, pancreatic secretions contribute lipases and in some cases, bile salts are required for lipase activation [49–51]. Thus, it is possible that the primary source of lipase is not derived from the hindgut itself, but enters from other organs. Hagfish have a large gall bladder yet any biliary activation would be missed in this study, as we determined tissue activity rather than luminal activity wherein the biliary activation would occur. Since the zymogen granule cells are the equivalent of a hagfish pancreas, it is unlikely that pancreatic contributions contribute much in terms of lipase. However, lipase activity can be influenced by dietary components [29,52] and has a broad variability in substrate specificity [13]. This likely means there are additional lipases with activities that were not recorded with our single substrate assay and that the activities themselves could change with diet.

Protease activity has been previously demonstrated in the Atlantic hagfish (*M*. *glutinosa*;[11,39]) and a broad spectrum of proteases is unsurprising given the carnivorous feeding habits of hagfish. While a different suite of proteolytic enzymes was examined, we too observed an unchanging activity along the length of the hindgut (Figs 4–6). Trypsin activity was only detected in the hindgut regions and did not appear to differ with nutritional status (Fig 4). Trypsin is released from the pancreas and is then restricted to the duodenum of vertebrates [51,53]. As mentioned above, the hagfish zymogen granules are likewise restricted to the hindgut and have been likened to pancreatic acinar cells, which could explain the hindgut restriction observed here. Alternatively, the lack of differentiation along the hagfish hindgut may lead to the absence of hindgut compartmentalization of function in favour of maximizing nutrient uptake along the entire tract length.

The activity of aminopeptidase was significantly elevated in the PCD region (Fig 5). Aminopeptidase is a primary brush border enzyme that is anchored in the plasma membrane of the vertebrate small intestine [54], which perhaps accounts for the unchanging activities found with feeding state. Furthermore, aminopeptidases are also involved in numerous functions including the initiation of a peptide anti-inflammatory response. Peptidases involved in such a response have been localized to the mammalian nasal passage [55], which is morphologically similar to the PCD in hagfish. Moreover, the other examined proteases were restricted to the hindgut region perhaps indicating that initial digestion could occur *via* aminopeptidase in the anterior tract.

Finally, we investigated alkaline phosphatase activity, which was also restricted to the hindgut, and may be utilized to demarcate between functional units of the intestine as previous [56]. Feeding resulted in a significant reduction of tissue alkaline phosphatase activity (Fig 6). Similarly to amylase and trypsin, alkaline phosphatase activity was found solely and consistently along the hindgut region, indicative of being stored within the zymogen granule cells. Interestingly, alkaline phosphatase is implicated in the uptake of glucose and lipids [57,58]. While most teleosts have an increased expression in the apical section of the intestine where nutrient uptake is elevated [41,49], Pacific hagfish have consistent alkaline phosphatase expression along the hindgut, which correlates with the unaltered uptake rates of both glucose [34] and lipids [47]. Alkaline phosphatase has been localized to both the brush border and enterocyte cytoplasm [59] and plays many roles in the intestine including pH regulation, fat acquisition, anti-inflammatory responses, as well as the potential regulation of the gut microbiome [16,60]. However since we observe a significant decrease in activity post-feeding, it is likely that our measurements relate to feeding in some way. Whether this is for digestion of the incoming meal or perhaps a more indirect role, such as gut mucosal defence [61], remains uncertain. Such mucosal defences may be of particular import for hagfish when they feast upon dead and decaying matter.

Interestingly, the lack of effect/ decrease in tissue protease activity with feeding contrasts a recent report of peptidase activity in the New Zealand hagfish, whereby feeding elicited an increase in peptidase activity in the mid and posterior regions of the hindgut [39]. This may be the result of differing substrates, which could indicate differential regulation of various proteases post-feeding and/or differences between the species themselves.

Given that the pH optima for these proteases fall in the alkaline range, we must consider the post-prandial acidification of the hagfish lumen [11,17]. We characterized each enzyme using a single pH value and the possibility exists that different activities could result if the pH was altered. Nilsson and Fänge (1970) [11] demonstrated a biphasic response of protease activity to changing pH. A strong proteolytic activity was observed at each of pH 4 and pH 9. If the animals do have a luminal acidification, does it persist along the entire length of the digestive tract? Is there a transition from acidic to alkaline in a time-dependent manner? These, among other questions, should be investigated in order to have a holistic understanding of hagfish post-prandial physiology.

### Environmental influences on digestive enzyme activity

The current viewpoint of digestive enzyme physiology suggests that activity correlates well with feeding ecology [62]. The suite of enzymes reflects the opportunistic feeding habits of hagfish and their ability to utilize a wide range of nutrients efficiently. Additionally, we observed rapid release of some enzymes following feeding (2 h post-feeding), which perhaps relates to the opportunistic feeding lifestyle employed by the hagfishes. Despite the potential for infrequent meals and an elevated tolerance to extended periods of fasting (>11 months; [46]), the food transit rate is fairly rapid and many physiological parameters affected by feeding return to resting rates by 12–48 h depending on species [17,39]. Therefore, the rapid rate of digestive enzyme release, likely from the zymogen granule cells, may be a consequence of intermittent and opportunistic feeding. Since most digestive enzymes can accept multiple substrates, the relative contributions of each type of enzyme (carbohydrase, lipase, protease) cannot be conclusively determined from this study. Throughout this study we utilized a single food source (squid). It is very likely that enzyme activity will vary with diet however we predict that the trends would remain constant with our observations. For example, those enzymes with a hindgut restriction and decreasing activity post-feeding are likely derived from the zymogen granule cells and thus, should continue to demonstrate reduced post-prandial activity irrespective of diet composition. The mechanism by which zymogen granules are released remains unknown. Despite vagal innervation, electrical stimulation did not yield any changes to gastric fluid production [11], and it is possible that a mechanical/stretch stimulus or a hormonal cue is responsible for granule release. Numerous hormones relating to feeding have been identified in the hagfish. For example, cholecystokinin is responsible for gall bladder contraction and pancreatic enzyme release in mammals yet its only confirmed role in hagfish is the activation of intestinal lipase secretion [63]. While a number of anti-sera have been investigated in hagfish species (gastrin, secretin, vasoactive intestinal peptide, substance P; [64–66]), our knowledge of the actions of these hormones as they pertain to feeding, digestion, or nutrient assimilation is very limited.

Diet composition can impact enzyme affinity and regulation, but can also induce changes to the gut microbiome. Bacteria are often associated with an organism’s digestive system and contribute to the success of nearly all studied animals. Digestive activity increases in regions of the digestive tract where the microbes are most densely populated [67,68] and contribute to the overall digestion within an animal. Yet, the microbiome itself is often a relatively unconsidered source of enzymatic activity, and is thus far unstudied in the hagfish. As mentioned above, a mucosal defense strategy within the hagfish digestive tract is likely important owing to their feeding behaviours. The alkaline phosphatase we detected along the hindgut may simultaneously inactivate bacterial pathogens, while recruiting commensal bacteria [61]. The hagfish gut microbiome constituents must either tolerate periodicity of feeding events or there will be a general turnover of the community depending on duration of fast or dietary composition.

Differences in digestive enzyme activity have also been attributed to circadian rhythms. For instance, in a sea cucumber species, rates of α-amylase and pepsin activity were elevated during times when this species is most active. Likewise, some fish species have shown diurnal rhythms of maximal digestive enzyme activity that coincides with feeding cycles [69]. We conducted our trials at the same time of day to ensure we would avoid differences induced by such rhythms. Hagfish are nocturnal and it is possible that our results underestimate maximal enzyme activity. However, we hypothesize that those enzymes that exist as zymogen granules will not change with time, as it is a stimulus-induced release rather than a membrane-bound protein with the possibility for up-regulation or altered affinity. We believe this is supported by the fact that hagfish can persist for many months without feeding and it would be a futile effort to continually alter enzyme activity and/or production. The Japanese hagfishes, *Eptatretus burgerii*, have a demonstrable seasonal migration [70] and may therefore, display a more regulated rhythmicity of digestive enzyme activity.

This experiment has quantified an array of digestive enzyme activity in the Pacific hagfish, comparable to their varied diet and metabolic requirements. Contrary to previous reports, digestive activity is observed along the entire length of the digestive tract. However, the majority of enzymes function within the hindgut region of the alimentary canal where absorption is prominent. The variable expression of these enzymes along the tract may be the first indications of compartmentalization of gut function. Although there is an obvious difference between the anterior and posterior tract in terms of cellular morphology, this is the first time that a physiological function other than lubrication is shown in the anterior portions. Functional differentiation along the hindgut is unlikely as there were no observed differences in activity along the length of the hindgut. As previously hypothesized, this likely permits a maximization of digestive function and nutrient assimilation across a relatively short digestive tract [30].

## Supporting information

S1 Fig Diagram depicting the various regions of the hagfish alimentary canal. PCD—pharyngocutaneous duct.
(PDF)

S1 Table Summary of statistics for Kruskal-Wallis comparisons between the anterior (B and PCD) and posterior (HG1-3) segments of the hagfish alimentary canal. (PDF)

S2 Table Summary of statistics for 2-way comparisons along the length of the hagfish alimentary canal and with differing feeding states. (PDF)
