# Supplementary material for: Activity and post-prandial regulation of digestive enzyme activity along the Pacific hagfish (Eptatretus stoutii) alimentary canal
Source: PLoS One. 2019 Apr 5;14(4):e0215027. doi: 10.1371/journal.pone.0215027 (PMC6450612; doi:10.1371/journal.pone.0215027)

**S1 Fig.** Diagram depicting the various regions of the hagfish alimentary canal. PCD – pharyngeocutaneous duct.

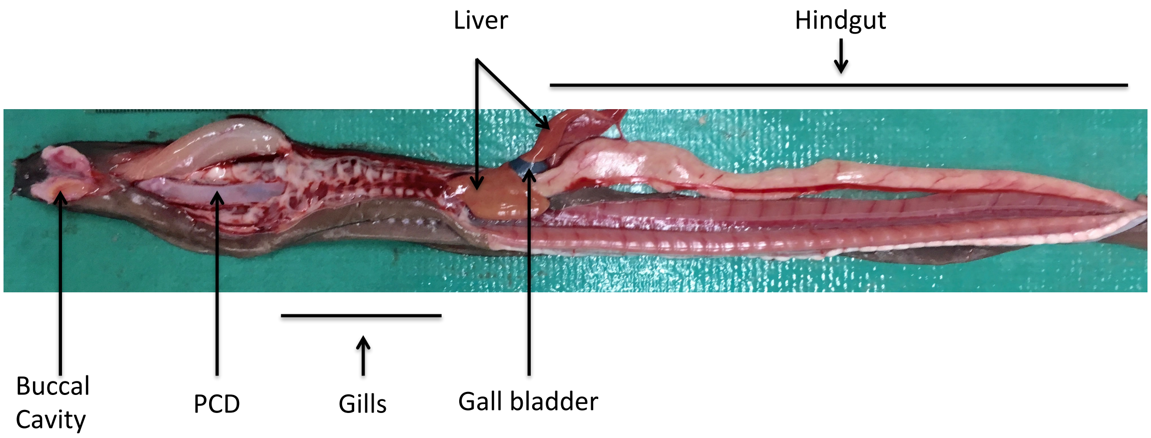

Supplement: S1 Fig — PCD—pharyngocutaneous duct. (PDF) [file pone.0215027.s001.pdf]
