# Supplementary material for: Activity and post-prandial regulation of digestive enzyme activity along the Pacific hagfish (Eptatretus stoutii) alimentary canal
Source: PLoS One. 2019 Apr 5;14(4):e0215027. doi: 10.1371/journal.pone.0215027 (PMC6450612; doi:10.1371/journal.pone.0215027)
Supplement: S1 Table — (PDF) [file pone.0215027.s002.pdf]

S1 Table. Summary of statistics for Kruskal-Wallis comparisons between the anterior (B and PCD) and posterior (HG1-3) segments of the hagfish alimentary canal

Enzyme	Statistical Summary
Amylase	$H_1 = 49.4$<br>$P < 0.001$
Maltase	$H_1 = 3.109$<br>$P = 0.078$
Lipase	$H_1 = 4.11$<br>$P = 0.043$
Trypsin	$H_1 = 58.407$<br>$P < 0.001$
Aminopeptidase	$H_1 = 1.398$<br>$P = 0.237$
Alk. Phosphatase	$H_1 = 68.411$<br>$P < 0.001$
