# Supplementary material for: Activity and post-prandial regulation of digestive enzyme activity along the Pacific hagfish (Eptatretus stoutii) alimentary canal
Source: PLoS One. 2019 Apr 5;14(4):e0215027. doi: 10.1371/journal.pone.0215027 (PMC6450612; doi:10.1371/journal.pone.0215027)
Supplement: S2 Table — (PDF) [file pone.0215027.s003.pdf]

S2 Table. Summary of statistics for 2-way comparisons along the length of the hagfish alimentary canal and with differing feeding states

Enzyme	Location	Feeding	Interaction
Amylase	$F_{2,40} = 2.621$<br>$P = 0.087$	$F_{1,40} = 56.0$<br>$P < 0.001$	$F_{2,40} = 1.568$<br>$P = 0.222$
Maltase	$F_{4,62} = 0.926$<br>$P = 0.099$	$F_{1,62} = 2.826$<br>$P = 0.099$	$F_{4,62} = 1.092$<br>$P = 0.370$
Trypsin	$F_{2,34} = 0.675$<br>$P = 0.517$	$F_{1,34} = 2.190$<br>$P = 0.149$	$F_{2,34} = 2.314$<br>$P = 0.116$
Lipase – post.	$F_{2,50} = 0.642$<br>$P = 0.531$	$F_{1,50} = 1.172$<br>$P = 0.285$	$F_{2,50} = 2.202$<br>$P = 0.122$
Aminopeptidase	$F_{4,79} = 10.791$<br>$P < 0.001$	$F_{1,79} = 0.0036$<br>$P = 0.953$	$F_{4,79} = 0.0379$<br>$P = 0.997$
Alk. Phosphatase	$F_{2,45} = 0.108$<br>$P = 0.898$	$F_{2,45} = 6.643$<br>$P = 0.014$	$F_{2,45} = 0.171$<br>$P = 0.843$
